# The Natural History and Management of Hepatic Hemangioma

**DOI:** 10.3390/jcm12175703

**Published:** 2023-09-01

**Authors:** Shigeo Maruyama, Tomomitsu Matono, Masahiko Koda

**Affiliations:** 1 Maruyama Medical Clinic, Aioimachi 3921, Hamada 697-0034, Shimane, Japan; shigeo.maruyama0304@gmail.com; 2 St. Mary’s Hospital, Nibuno 650, Himeji 670-0801, Hyogo, Japan; matomato@himemaria.or.jp; 3 Hino Hospital, Nota 332, Hino 689-4504, Tottori, Japan

**Keywords:** hepatic hemangioma, coagulation disorder, liver fibrosis, natural history, management

## Abstract

Background: Knowledge of the natural history and management of hepatic hemangiomas is lacking. The aim of this study was to investigate the natural history of hemangiomas and to elucidate the factors that determine tumor growth and optimal management. Methods: A total of 211 adult patients were enrolled, with follow-up for more than three years. Follow-up was performed with repeated ultrasonography (US) and laboratory tests for liver function and coagulation factors (platelets, prothrombin time (PT), fibrinogen, thrombin–antithrombin III complex (TAT), D-dimer, and fibrin and fibrinogen degradation products (FDP)). Results: Tumor size decreased in 38.9% of patients, showed no change in 31.3%, and increased in 29.8%. The incidence of a size increase was very high in patients under 40 years of age and decreased gradually with age, whereas the incidence of a size decrease increased with age and increased markedly over 60 years of age. The incidence of an increase in size decreased gradually with size enlargement, whereas the incidence of a decrease in size increased markedly with tumor size and further increased rapidly when hemangiomas became larger than 60 mm. Values of TAT, D-dimer, FDP, and Mac-2 binding protein glycosylation isomer (M2BPGi) were closely related to the change in size of hemangiomas. Conclusions: Hemangiomas in older patients (>60 years of age) and larger tumors (>60 mm in size) had a tendency to decrease in size, resulting from the reduction in coagulation disorders and the progression of liver fibrosis. Therefore, the majority of patients with hemangiomas can be safely managed by clinical observation.

## 1. Introduction

Hepatic hemangiomas are the most common benign tumors of the liver. Most hemangiomas are small and asymptomatic; therefore, these tumors have little clinical significance and require no treatment. In view of this, little is known of the natural history and growth pattern of these tumors, and their appropriate management is still not clearly defined. The main reason for this is that previous studies have not shown sufficient data on the natural history of hemangiomas and on their tendency to change in size or to cause complications [1,2]. We previously reported that differences in tumor size were caused by intratumoral thrombosis and subsequent hemorrhage [2] and further elucidated the predictive risk factors for hemangioma-related complications [3]. The aims of this study were to investigate the natural history and growth pattern of hemangiomas by monitoring sonographic changes in size, alongside clinical factors, especially coagulation factors and Mac-2 binding protein glycosylation isomer (M2BPGi), which has been identified as a useful biomarker for assessment of liver fibrosis; to elucidate the factors that determine tumor growth; and to clarify the optimal management of patients with hemangiomas.

## 2. Materials and Methods

### 2.1. Patients

This study was approved by the ethics review board of Tottori University Hospital (approval number: 18A023) and the ethics committee of Hino Hospital (approval number: 2018-4). Of 26,871 abdominal ultrasonography (US) examinations performed at our hospital, Hino Hospital, and Tottori University Hospital, Japan, between January 2016 and December 2022, a total of 321 patients were diagnosed with hepatic hemangiomas and were consecutively enrolled in this study after providing their informed consent. Of the 321 patients initially diagnosed with hepatic hemangiomas, 211 patients with a follow-up period of at least three years were retrospectively enrolled in this follow-up study. Patients with infectious diseases, liver cirrhosis, or malignant tumors were excluded from the study. Patients with abnormal values of thrombin–antithrombin III complex (TAT) (>3.0 ng/mL), D-dimer (>1.0 µg/mL), and fibrin and fibrinogen degradation products (FDP) (>5.0 µg/mL) were referred for cardiovascular medicine consultation to prevent complications of vascular thrombotic diseases. No patients were treated with anticoagulation therapy.

### 2.2. Methods

Hepatic hemangiomas were diagnosed by US and multiphase contrast-enhanced helical computed tomography (CT). Their appearance on US comprised homogeneous, hyperechoic, well-defined lesions, although larger hemangiomas showed mixed echogenicity. On CT, they were characterized as well-defined, homogeneous, hypodense lesions with peripheral nodular enhancement followed by progressive centripetal enhancement [1,2,3]. In most hemangiomas (diameter ≤ 20 mm), the final diagnosis was based on US features, clinical observation, and negative biochemical results. Relatively large lesions (diameter > 20 mm) were diagnosed using both US and CT. Abdominal US was performed in all patients, and CT was performed in 114 patients. All patients were examined by US examiners who had 30 years of experience.

Follow-up was performed with repeated US examinations and laboratory tests for liver function and coagulation factors (platelets, prothrombin time (PT), fibrinogen, TAT, D-dimer, and FDP) every 3–6 months at the time of regular medical examination or annually at periodic health checkups. The standard rate of change in tumor size was established, and the changes in size were classified into three groups according to the rate of change: (1) decrease, change ≤−10%; (2) no change, change >−10% to <+10%; and (3) increase, change ≥+10%. At least three transverse, longitudinal, and anteroposterior measurements were taken of each lesion. When none of the three diameters coincided with the maximum diameter of the hemangioma, a section containing the maximum diameter was measured [4] and it was used for rate of change in tumor size. The changes in tumor size within upper and lower limits of 2 mm were not considered important due to the measurement limitations of US [4]. Using the categories described in a previous study [5], hepatic hemangiomas were divided into three groups according to their maximum diameter: small (<20 mm), medium (20–40 mm), and large groups (>40 mm).

### 2.3. Statistical Analysis

All measurements are expressed as means ± standard deviation (SD). Significant differences among two or three groups were determined using Student’s *t*-test or one-way analysis of variance with Kruskal–Wallis of Dunn’s post hoc test. Categorical variables were analyzed using the chi-squared test. The data were analyzed using Stat Flex version 6.0 (Artech Co., Ltd., Osaka, Japan). A *p* value < 0.05 was considered significant.

## 3. Results

### 3.1. Characteristics of Patients and Hemangiomas

Among the 216 patients with hepatic hemangiomas, 5 patients who had high values of protein induced by vitamin K absence or antagonist-II (PIVKA-II) were excluded because differentiation from malignant tumors was required. A total of 211 patients were enrolled in the present study. Laboratory findings at the first examination in 211 patients with hepatic hemangiomas are shown in Table 1. There were 77 men and 134 women (male:female, 1:1.7), with a median age of 57 years (range, 23–89 years). The median follow-up period was 56.5 months (range, 36–81 months). The median size of hemangiomas was 22.1 mm (range, 5.1–107 mm). Table 2 shows the relationship between tumor size and clinical parameters in these patients. The mean age was significantly higher in the large group than in the other groups (*p* < 0.05). Hemoglobin concentration was significantly lower in the large group than in the other groups (*p* < 0.05). Platelet counts and fibrinogen levels were significantly lower in the large group than in the other groups (*p* < 0.05). Values of TAT, D-dimer, FDP, and M2BPGi were significantly higher in the large group than in the other groups (all *p* < 0.0001).

### 3.2. Relationship between Changes in Hemangioma Size and Clinical Parameters

During the follow-up period, none of the patients developed any complications correlated with the hemangioma, received surgical treatment, or dropped out of the study, with all 211 patients completing this study. Table 3 shows the relationship between changes in tumor size and clinical parameters in 211 patients with hepatic hemangiomas. The mean age and follow-up period were significantly higher in the decrease group than in the other groups (*p* < 0.0001 and *p* < 0.01, respectively). Albumin was significantly lower, and alkaline phosphatase (ALP) was significantly higher in the decrease group than in the other groups (*p* < 0.01 and *p* < 0.05, respectively). Platelet counts were significantly lower in the decrease group than in the other groups (*p* < 0.01). TAT (*p* < 0.001), D-dimer (*p* < 0.0001), FDP (*p* < 0.001), and M2BPGi levels (*p* < 0.001) were significantly elevated in the decrease group. Tumor size was significantly larger in the decrease group than in the no-change and increase groups (both *p* < 0.0001), which can be considered to reflect the significant difference in platelet counts and values of TAT, D-dimer, and FDP among the three groups.

### 3.3. Ultrasonographic Follow-Up

During the follow-up period, 38.9% (82/211) of patients had hemangiomas that decreased in size, with a median decrease of 8.2 mm (range, 2.1–42.5 mm); 31.3% (66/211) had tumors with no change in size; and 29.8% (63/211) had tumors that increased in size, with a median increase of 5.8 mm (range, 2.1–36.2 mm). The tendency of a tumor to decrease or increase size persisted in the decrease and increase groups, respectively, without change during the observational period. It is interesting that 7 (3.3%) patients in the decrease group had hemangiomas that disappeared.

**Figure 1:**
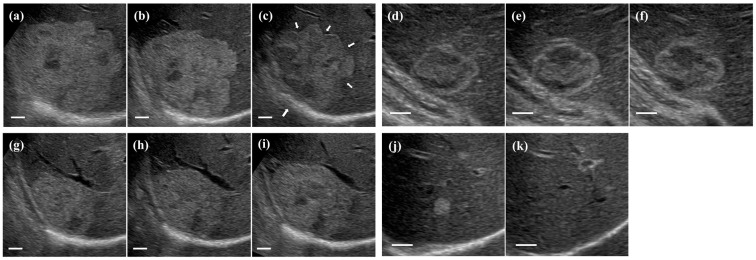
Sonographic changes in size of hepatic hemangiomas. (**a**–**c**) Longitudinal sonograms of a hepatic hemangioma in a patient with decreased tumor size. The initial sonogram (**a**) and follow-up sonograms obtained 39 (**b**) and 81 months (**c**) after the initial diagnosis. Retraction of the liver capsule is seen adjacent to the tumor (large arrow), and concavity of the tumor margin becomes more marked with time (small arrow). (**d**–**f**) Longitudinal sonograms of a hepatic hemangioma in a patient with no change in tumor size. The initial sonogram (**d**) and follow-up sonograms obtained 33 (**e**) and 77 months (**f**) after the initial diagnosis. (**g**–**i**) Longitudinal sonograms of a hepatic hemangioma in a patient with increased tumor size. The initial sonogram (**g**) and follow-up sonograms obtained 42 (**h**) and 79 months (**i**) after the initial diagnosis. (**j**,**k**) Longitudinal sonograms of a hepatic hemangioma in a patient in whom the tumor disappeared. The follow-up sonogram obtained 79 months (**k**) after the initial diagnosis shows no evidence of the hemangioma in the previously noted region (**j**). White bars indicate a length of 10 mm.

Figure 1 shows sonographic changes in size of hepatic hemangiomas. Figure 1a–c show longitudinal sonograms of a 61-year-old man with a decrease in size. The initial sonogram (a) showed a hemangioma with a diameter of 80.8 mm. Follow-up sonograms obtained 39 (b) and 81 months (c) after the initial diagnosis showed decreases in diameter to 71.2 and 60.6 mm, respectively. Retraction of the liver capsule was seen adjacent to the tumor (large arrow), and concavity of the tumor margin became more marked with time (small arrow). Figure 1d–f show longitudinal sonograms of a 52-year-old woman with no change in size. The initial sonogram (d) showed a hemangioma with a diameter of 36.3 mm. Follow-up sonograms obtained 33 (e) and 77 months (f) after the initial diagnosis showed no changes in diameter (36.5 mm and 37.2 mm, respectively). No changes were observed in the internal echo pattern or tumor margin. Figure 1g–i show longitudinal sonograms of a 47-year-old man with an increase in size. The initial sonogram (g) showed a hemangioma with a diameter of 49.2 mm. The follow-up sonograms obtained 42 (h) and 79 months (i) after the initial diagnosis showed increases in diameter to 58.3 mm and 66.7 mm, respectively. The hypoechoic area was observed to increase with size enlargement. Figure 1j,k show longitudinal sonograms of a 74-year-old woman in whom the tumor disappeared. The initial sonogram (j) showed a hemangioma with a diameter of 10.8 mm. The follow-up sonogram obtained 79 months (k) after the initial diagnosis showed no evidence of the hemangioma in the previously noted region.

### 3.4. Comparison of Clinical Parameters at the First Examination with Those at the Last Examination in the Follow-Up Period by Change in Hemangioma Size

Table 4 shows the comparative values between the first and last examinations among each group in terms of changes in size in 211 patients with hepatic hemangiomas. In the decrease group, values of TAT (*p* < 0.05), D-dimer (*p* < 0.0001), and FDP (*p* < 0.001) were significantly lower, and M2BPGi levels (*p* < 00001) were significantly higher at the last examination than at the first examination. In the no-change group, values of all coagulation markers and M2BPGi were not significantly different between the first and last examinations. In the increase group, platelet counts (*p* < 0.01) and fibrinogen levels (*p* < 0.05) were significantly lower, and values of TAT (*p* < 0.05), D-dimer (*p* < 0.001), and FDP (*p* < 0.05) were significantly higher, whereas values of M2BPGi (*p* < 0.05) were significantly lower at the last examination than at the first examination.

### 3.5. Growth Pattern of Hepatic Hemangiomas

**Figure 2:**
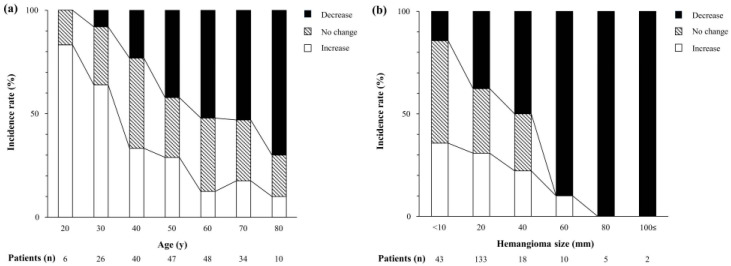
Incidence rate of hepatic hemangiomas and growth patterns. (**a**) Rate and pattern according to patient age and (**b**) tumor size.

Figure 2 shows the change in the incidence of each growth pattern of hepatic hemangiomas. Figure 2a shows the change in the incidence by patient age. In patients <40 years of age, the incidence of an increase in size was comparatively high and decreased gradually with age, whereas the incidence of a decrease in size increased gradually with age and increased markedly over 60 years of age. The incidence of no change in size showed similar trends in all age groups. These results demonstrate that hemangiomas in patients <40 years of age had a tendency to increase in size, and those in patients >60 years of age had a tendency to decrease in size. Figure 2b shows the change in the incidence by tumor size. The incidences of increase and no change in size decreased gradually with size enlargement. In contrast, the incidence of a decrease in size increased markedly with tumor size and further increased rapidly when the hemangiomas became greater than 60 mm. These results demonstrate that hemangiomas have a tendency to decrease in size with size enlargement, especially with a tumor size >60 mm.

### 3.6. Effects of Age, Liver Disease, and Hemangioma Size on the Changes in Values of M2BPGi

The effects of patient age, presence of liver disease, and hemangioma size on the changes in values of M2BPGi were investigated. Table 5 shows values of M2BPGi in the different age groups under the specified categories in 321 patients initially diagnosed with hepatic hemangiomas. Patients were classified by age into three groups (>60 years, 40–60 years, and <40 years), excluding patients with chronic liver disease as the underlying disease, who were classified into two groups (>60 years and ≤60 years, since there were no patients aged <40 years). Values of M2BPGi were significantly correlated with age in the 321 patients with hemangiomas (*p* < 0.0001) (data not shown). In all 321 patients and 274 patients without chronic liver disease, values of M2BPGi were significantly elevated with increased age (*p* < 0.0001 and *p* < 0.001, respectively). Among 47 patients with chronic liver disease, values of M2BPGi in patients aged >60 years were slightly but not significantly higher than those in patients aged ≤60 years. These results demonstrate that values of M2BPGi were elevated with increases in age, regardless of the existence of chronic liver disease. Values of M2BPGi were significantly correlated with tumor size in the 321 patients with hemangiomas (*p* < 0.0001) (data not shown). In 211 patients with small hemangiomas, values of M2BPGi were significantly elevated with increased age (*p* < 0.0001). In 83 and 27 patients with medium and large hemangiomas, respectively, values of M2BPGi were not significantly different among the three groups. However, values of M2BPGi were significantly higher in large hemangiomas than in small and medium hemangiomas, regardless of age (data not shown). These results demonstrate that the values of M2BPGi were elevated with increased age and tumor size, although the elevation in M2BPGi was less affected by age than by tumor size, along with hemangioma enlargement.

## 4. Discussion

The natural history and growth pattern of hepatic hemangiomas are not well understood. Several studies have examined the natural course of hemangiomas, and they reported that the vast majority remained stable in size [1,4,6,7]. There is general agreement that hemangiomas increase in size following intratumoral thrombosis or hemorrhage [1,2,8]. In contrast, some authors have reported that hemangiomas might undergo degeneration and fibrous replacement, which would explain the size decrease in hemangiomas [1,8]. Few data have been reported to elucidate the change in hemangioma size, the main reason for this paucity being the limited availability of data on the natural history of hemangiomas.

Kasabach–Merritt syndrome (KMS) is a well-known condition involving giant hepatic hemangioma characterized by thrombocytopenia and consumption coagulopathy [8,9]. Consumption of platelets and coagulation factors in addition to ongoing fibrinolysis results in intralesional bleeding and enlargement of hemangiomas; consequently, the hemangiomas may lead to KMS [10]. KMS is considered the most extreme stage of giant hemangiomas and the most representative as a type of hemangioma that increases in size. In contrast, hepatic hemangiomas can undergo regressive changes such as thrombosis, necrosis, and scarring [9]. Tumors that undergo degeneration and fibrous replacement are called sclerosed hemangiomas (SHs) [11]. SHs are considered to be the end stage of the process of tumor growth and a rare type of hemangioma [12]. Goodman reported that hepatic hemangiomas and SHs were the same kinds of tumors presenting at different times of almost the same natural history [13]. SHs are considered the most representative type of hemangioma that decreases in size. Consequently, it can be said that KMS and SHs are located in the extreme stage of tumor growth in the natural history of hemangiomas. In other words, hepatic hemangiomas are located between KMS, which is known for coagulopathy, and SHs, which characterized by fibrosis and hyalinization, in the process of tumor growth.

We previously investigated the correlations between hemangioma size and coagulation factors and concluded that tumor size was associated with coagulation factors, especially TAT, D-dimer, and FDP, which were considered to be useful for the diagnosis of coagulopathy [2,3]. In our follow-up study, TAT, D-dimer, and FDP levels were significantly lower at the last examination than at the first examination in patients with decreases in size, whereas in patients with increases in size, they were significantly higher at the time of the last follow-up. Based on these results, we can hypothesize that coagulation factors play a part in the growth of hemangiomas and that the size increase in hemangiomas is closely associated with coagulation disorders.

Hemangiomas undergo degeneration and fibrous replacement within the tumor [8,9] and change into varying degrees of fibrosis with age through the regression process of tumors [5,8]. Fibrosis noted around hemangiomas was associated with regional circulatory disorders in the peripheral liver triggered by thrombus formation within tumors [14] and blood flow disturbances caused by the compression effect exerted by the tumor with an increase in size [15]. In a case study, some authors reported the existence of hepatic atrophy or uneven morphological changes around hemangiomas and concluded that these changes were mainly caused by regional circulatory disorders, along with the progression of fibrosis [16]. In the present study, retraction of the liver capsule and concavity of the tumor margin were observed in some patients with decreases in size, and this concavity became more marked with a decrease in size (see Figure 1c). Capsular retraction adjacent to hemangiomas is due to necrosis and fibrosis within the tumor, which is seen more often in larger hemangiomas [17]. We suggest that the retraction of the liver capsule and concavity of the tumor margin seen in the presented cases are morphological changes caused by the progression of fibrosis within and surrounding tumors. Fibrous degeneration and replacement within hemangiomas and progressive and extensive fibrosis surrounding tumors resulted in focal fibrosis of the liver, which is hereafter referred to as “hemangioma-related fibrosis” in the present study.

It is widely acknowledged that moderate fibrosis is a histological hallmark of an aging liver. Bos et al. reported that the metabolism of collagen was reduced with aging in normal subjects [18], and other authors reported that the collagenolytic activity of matrix metalloproteinase (MMP) was reduced and liver fibrosis developed in an aging rat [19]. Therefore, it appears that collagenolytic activity decreases, and, subsequently, hepatic fibrosis increases in the liver of elderly persons [20]. The progression of liver fibrosis with aging is hereafter referred to as “age-related fibrosis” in the present study. The presented results demonstrated that liver fibrosis progressed with age, although fibrosis progression was less affected by age than by tumor size, along with hemangioma enlargement. Therefore, liver fibrosis associated with hemangiomas appears to consist of both hemangioma-related fibrosis and age-related fibrosis, and the former might play a more significant role in the progression of liver fibrosis than the latter, along with increases in age and tumor size.

M2BPGi has been identified as a reliable and non-invasive marker for assessment of liver fibrosis [21]. Yamasaki et al. found that M2BPGi correlated with the stage of fibrosis, platelets, albumin, total bilirubin, alanine aminotransferase (ALT), and age and served as a reliable marker for assessing liver fibrosis [21,22]. The present results also demonstrate that M2BPGi levels correlated with platelets (*p* < 0.0001), PT (*p* < 0.05), albumin (*p* < 0.0001), ALT (*p* < 0.0001), and age (*p* < 0.0001) (data not shown), which were test items related to liver fibrosis, suggesting that the elevation in M2BPGi might be caused by the development of liver fibrosis consisting of both hemangioma-related fibrosis and age-related fibrosis. It is widely acknowledged that M2BPGi levels are closely related to the fibrosis stage of chronic liver disease [22,23]. In fact, in the present study, values of M2BPGi were significantly higher in than 47 patients with chronic liver disease as the underlying disease than in the 274 patients without chronic liver disease (*p* < 0.01) (data not shown). However, mean M2BPGi values were 0.73 and 0.51 COI in patients with and without chronic liver disease, respectively, which are within the range [21,22], suggesting that the elevation of M2BPGi seen in the present study might not be pathologically significant compared to the degree of fibrosis in patients with chronic liver disease and liver fibrosis. The present results relating to the change in M2BPGi values by tumor growth demonstrate that the elevation of M2BPGi was found in patients with decreases in size, no change in patients with stable lesions, and a decrease in patients with increases in size. Based on the present results, we can speculate that liver fibrosis plays a part as a growth-inhibitory factor for hemangiomas and that the size decrease in hemangiomas is closely associated with liver fibrosis, mainly with respect to hemangioma-related fibrosis.

A recent study emphasized that hemangiomas increase in size for a certain period of time and might ultimately become stable or decrease in size, also showing that the peak growth period of hemangiomas was in patients <30 years of age and that the growth rate decreased rapidly after 50 years of age. In addition, the growth rate increased up to a tumor size of 10 cm, then decreased rapidly when the hemangiomas became larger than 10 cm [1]. The present study shows that the high-growth period of hemangiomas occurred when patients were <40 years of age, and when patients were older than 60 years of age, the growth tendency decreased rapidly, with the decreasing tendency becoming markedly more pronounced with increased age. Moreover, the growth tendency decreased gradually with size enlargement, whereas the decreasing tendency increased markedly with tumor size and further increased rapidly when the hemangiomas became larger than 60 mm. These results suggest that the growth patterns were different depending on patient age and hemangioma size, but relatively younger (<40 years) patients had a tendency to show increases in size, and hemangiomas in older (>60 years) patients and larger tumors (>60 mm in size) had a tendency to show decreases in size.

Seven patients were identified in the follow-up study in whom the hemangioma had disappeared. In all of these patients, CT was performed to confirm the disappearance of the tumor. The characteristic findings were as follows: (1) patients were relatively old (mean age 75, years); (2) tumors were small (size, ≤20 mm; mean size, 14.0 mm); (3) values of TAT, D-dimer, and FDP were all normal at both the first and last examinations; and (4) mean M2BPGi values at both times (0.98 and 1.46 COI, respectively) were relatively high—comparable to fibrosis grades F0-1 and F1-2 [24,25], respectively. Based on the above findings, the disappearance of hemangiomas appeared to occur in elderly patients with progression of fibrosis in the liver and small hemangiomas unaccompanied by coagulation disorders.

Based on these research results, we suggest that hepatic hemangiomas grow gradually with time, and subsequently, tumors change in size as a result of some mechanisms in the process of tumor growth. The change in size of hemangiomas depends on the balance between the growth factors caused by coagulation disorders and the growth-inhibitory factors caused by liver fibrosis. Coagulation disorders are caused by consumption of platelets and coagulation factors triggered by thrombus formation within the vessels of hemangiomas [2,3], while these vessels are degenerated by the progression of fibrosis and subsequent regional circulatory disorders through the regression process of tumors [5,8,9,16], consequently resulting in the reduction in coagulation disorders. On the other hand, liver fibrosis progresses with age and tumor size. Therefore, the inhibitory factors may play a more significant role in the change in tumor size than the growth factors, along with increases in patient age and tumor size; consequently, the hemangioma size may be stable and ultimately decrease.

The optimal management of patients with hepatic hemangiomas remains controversial. While there is general agreement that small asymptomatic hemangiomas should be managed conservatively, the unknown natural history and the possibility of complications of hemangiomas make treatment selection difficult [26]. Although the indications for surgery include the presence of progressive abdominal pain or discomfort, rapid growth of the tumor, spontaneous rupture, KMS, and unclear diagnosis [27], the majority of patients with hemangiomas, except patients with KMS, which is considered the absolute indication for treatment [28,29], can be safely managed by clinical observation. Meanwhile, the absolute indications for surgery are still not clearly defined. The main reason for this is the limited availability of data on the natural history of hemangiomas and their tendency to cause complications. Therefore, this longitudinal study was performed to investigate such issues and elucidate the optimal management of patients with hemangiomas.

Progressive abdominal pain or discomfort is the most common indication for surgical treatment. Although the exact mechanism of pain or discomfort is unclear, increasing size and intratumoral thrombosis or hemorrhage could cause these symptoms as a result of liver capsule distension [27]. The present results show that hemangiomas decreased in size as a result of the reduction in coagulation disorders and the progression of liver fibrosis with patient age and tumor size; consequently, hemangiomas in older patients (>60 years of age) and larger tumors (>60 mm in size) showed a tendency to decease in size. A recent study demonstrated that the growth rate of hemangiomas decreased in patients after 50 years of age and in hemangiomas >10 cm in size; therefore, hemangiomas rarely cause complications, even when large in size, and the authors proposed that all hemangiomas can be safely managed by observation, even in patients with symptoms [1]. On the basis of our results and those recently reported in [1], we suggest that the incidence of abdominal symptoms caused by hemangiomas eventually decreases with patient age and tumor enlargement. Therefore, we propose that the vast majority of hepatic hemangiomas can be safely managed by observation until a patient age of 60 years and a hemangioma size of 60 mm, even in patients with abdominal symptoms.

Rapidly growing hemangiomas represent a further indication for surgical treatment, although it is difficult to predict progression to rapid growth. The size increase in hemangiomas is closely associated with intratumoral thrombosis or hemorrhage caused by coagulation disorders [2,3]. The present results demonstrate that the growth rate of hemangiomas decreased with increases in age and tumor size as a result of the reduction in coagulation disorders and the progression of liver fibrosis; therefore, the rapid growth of hemangiomas is to be considered an exceptional event. However, some authors have emphasized that hemangiomas >10 cm in size may have a greater potential for rapid growth triggered by internal bleeding caused by coagulation disorders [27], and others proposed that a minimum 25% increase in the largest diameter within a period of 6 months could be chosen to characterize rapidly growing hemangiomas [30]. For the management of patients with hemangiomas at risk of developing rapid growth, we emphasize that the follow-up tumor size measurement using US, as well as the evaluation of coagulation parameters, is indicated. Accordingly, hemangiomas more than 10 cm in size exhibiting a 25% or greater increase in maximum diameter within 6 months in patients with persistent coagulation disorders could be considered for surgical treatment.

Spontaneous rupture is a potentially life-threatening complication and an absolute indication for surgery. Some authors demonstrated that the risk of rupture was rare in hemangiomas ≤ 10 cm in diameter [6], and others recommended that preventive surgery should be considered for hemangiomas > 11 cm in size in special cohorts of patients, especially elderly patients (age > 65 years) [31]. Hypotheses to explain spontaneous rupture include rapid growth of the tumor leading to necrosis and coagulopathy. Although the exact risk of spontaneous rupture remains unknown, the risk of rupture is extremely low [31]. In fact, in the present study, no spontaneous rupture of hemangiomas was observed, even though large tumors ≥ 10 cm in size were present. Histological analysis has shown that hemangiomas are tenacious and not easily ruptured [1], and based on the present results, we can also conjecture that hemangiomas become more tenacious and sclerotic and even more difficult to rupture as a consequence of the reduction in coagulation disorders and the histologic sclerotic change caused by progressive fibrosis. Therefore, we believe that prophylactic operative treatment cannot be recommended, even in patients with relatively large hemangiomas. However, preventive surgery should be considered only for hemangiomas > 10 cm in size exhibiting a progressive increase in tumor size, especially in elderly patients (age > 65 years) [31] and/or patients with progressive and severe coagulopathy [10,26,32].

The present study has several limitations. First are the limitations regarding the length of follow-up and the number of patients with giant hemangiomas, as there were no cases requiring surgery due to hemangioma-related complications. Therefore, a longer follow-up period and a larger patient cohort with giant hemangiomas are needed to evaluate the optimal management of these tumors. Second, hemangiomas in the present cases were not surgically resected, and a pathological correlation was not available; therefore, further studies are required to evaluate the relationship between liver fibrosis consisting of hemangioma-related fibrosis and age-related fibrosis and the elevation of M2BPGi levels associated with hemangiomas.

## 5. Conclusions

The present study is the first to investigate the natural history and growth pattern of hepatic hemangiomas and elucidate the factors that determine tumor growth and optimal management. Hemangiomas in older patients (>60 years of age) and larger tumors (>60 mm in size) had a tendency to decrease in size. The change in size of hemangiomas depended on the balance between the growth factors that resulted from coagulation disorders and the growth inhibitory factors caused by liver fibrosis. The inhibitory factors might play a more significant role in the change in tumor size than the growth factors in the natural history. Consequently, hemangioma size might be stable and ultimately decrease. Therefore, the majority of hemangiomas can be safely managed by clinical observation, and treatment may be considered only for patients with a risk of spontaneous rupture or KMS.

## Figures and Tables

**Table 1:** Laboratory findings of 211 patients with hepatic hemangiomas.

Parameters		Value
Age (years)		57 ± 14
Male/female (*n*)		77/134
Follow-up period (months)		56.5 ± 14.5
Size of hemangioma (mm)		22.1 ± 17.8
Small	<20	123 (58.3%)
Medium	20–40	63 (29.9%)
Large	>40	25 (11.8%)
Location of hemangioma (*n*)		
Right lobe		184 (87.2%)
Left lobe		25 (11.8%)
Bilateral lobe		2 (1.0%)
Number of hemangiomas (*n*)		
Single		180 (85.3%)
Multiple		31 (14.7%)
Biochemistry		
Total bilirubin (mg/dL)		0.6 ± 0.2
Albumin (g/dL)		4.2 ± 0.2
ALT (U/L)		20 ± 13
GGT (U/L)		42 ± 51
ALP (U/L)		232 ± 72
BUN (mg/dL)		14.6 ± 3.7
Cr (mg/dL)		0.72 ± 0.18
Hematology		
Hemoglobin (g/dL)		13.6 ± 1.3
Platelets (10^4^/μL)		22.3 ± 5.5
Coagulation		
PT (%)		94.0 ± 12.8
Fibrinogen (mg/dL)		276 ± 67
TAT (ng/mL)		1.42 ± 1.06
D-dimer (μg/mL)		0.72 ± 0.69
FDP (μg/mL)		1.63 ± 1.00
Serology		
M2BPGi (COI)		0.61 ± 0.33
AFP (ng/mL)		3.5 ± 1.5
PIVKA-II (mAU/mL)		20.4 ± 5.8
Associated liver diseases (*n*)		
Hepatitis B		12 (5.7%)
Hepatitis C		5 (2.4%)
Autoimmune hepatitis		7 (3.3%)
Primary biliary cholangitis		2 (0.9%)
Alcoholic liver disease		16 (7.6%)
Nonalcoholic steatohepatitis		5 (2.4%)

All data represent values at the time of study enrollment. Values are presented as means ± standard deviation or numbers (%). ALT, alanine aminotransferase; GGT, γ-glutamyl transpeptidase; ALP, alkaline phosphatase; BUN, blood urea nitrogen; Cr, creatinine; PT, prothrombin time; TAT, thrombin–antithrombin III complex; FDP, fibrin and fibrinogen degradation products; M2BPGi, Mac-2 binding protein glycosylation isomer; AFP, α-fetoprotein; PIVKA-II, protein induced by vitamin K absence or antagonist-II.

**Table 2:** Association between tumor size and clinical parameters in 211 patients with hepatic hemangiomas.

Parameters	Small (*n* = 123)	Medium (*n* = 63)	Large (*n* = 25)	*p* Value
Age (years)	55 ± 15	57 ± 14	64 ±12	0.0309
Male/female (*n*)	41/82	27/36	11/14	0.4988
Biochemistry				
Total bilirubin (mg/dL)	0.6 ± 0.2	0.6 ± 0.3	0.6 ± 0.2	0.7739
Albumin (g/dL)	4.2 ± 0.2	4.3 ± 0.2	4.0 ± 0.3	0.0036
ALT (U/L)	21 ± 16	20 ± 10	16 ± 6	0.1880
GGT (U/L)	43 ± 59	43 ± 40	30 ± 29	0.1582
ALP (U/L)	230 ± 73	234 ± 68	236 ± 82	0.9203
BUN (mg/dL)	14.2 ± 3.5	14.6 ± 3.8	16.4 ± 3.8	0.0314
Cr (mg/dL)	0.71 ± 0.15	0.73 ± 0.22	0.77 ± 0.18	0.3940
Hematology				
Hemoglobin (g/dL)	13.7 ± 1.2	13.7 ±1.2	12.8 ± 1.7	0.0246
Platelets (10^4^/μL)	22.7 ± 5.2	21.6 ± 5.5	20.0 ± 6.5	0.0159
Coagulation				
PT (%)	93.0 ± 12.9	95.9 ± 12.8	95.2 ± 10.4	0.4029
Fibrinogen (mg/dL)	284 ± 66	276 ± 66	238 ± 63	0.0104
TAT (ng/mL)	1.11 ± 0.52	1.26 ± 0.62	3.36 ± 1.71	<0.0001
D-dimer (μg/mL)	0.50 ± 0.30	0.61 ± 0.34	2.06 ± 1.13	<0.0001
FDP (μg/mL)	1.33 ± 0.22	1.55 ± 0.63	3.33 ± 1.92	<0.0001
Serology				
M2BPGi (COI)	0.53 ± 0.27	0.67 ± 0.35	0.88 ± 0.32	<0.0001
AFP (ng/mL)	3.3 ± 1.5	4.3 ± 1.5	3.4 ± 1.8	0.0021
PIVKA-II (mAU/mL)	19.5 ± 5.5	20.8 ± 5.9	23.6 ± 6.4	0.0077
Ultrasound findings				
Echo pattern				<0.0001
Homogeneous type (*n*)	122 (99.2%)	27 (42.9%)	0 (0%)	
Mixed type (*n*)	1 (0.8%)	36 (57.1%)	25 (100%)	
Portal vein diameter (mm)	10.3 ± 2.0	11.2 ± 1.7	12.8 ± 1.9	<0.0001
Spleen index (mm^2^)	1220 ± 490	1259 ± 468	1702 ± 639	0.0013

All data represent values at the time of study enrollment. Values are presented as means ± standard deviation or numbers (%). ALT, alanine aminotransferase; GGT, γ-glutamyl transpeptidase; ALP, alkaline phosphatase; BUN, blood urea nitrogen; Cr, creatinine; PT, prothrombin time; TAT, thrombin–antithrombin III complex; FDP, fibrin and fibrinogen degradation products; M2BPGi, Mac-2 binding protein glycosylation isomer; AFP, α-fetoprotein; PIVKA-II, protein induced by vitamin K absence or antagonist-II.

**Table 3:** Associations between changes in tumor size and clinical parameters in 211 patients with hepatic hemangiomas at the first examination.

Parameters	Decrease (*n* = 82)	No Change (*n* = 66)	Increase (*n* = 63)	*p* Value
Age (years)	64 ± 11	55 ± 13	48 ± 14	<0.0001
Male/female (*n*)	27/55	23/43	27/36	0.5881
Follow-up period (months)	58.0 ± 10.9	52.8 ± 11.9	50.9 ± 10.5	0.0041
Biochemistry				
Total bilirubin (mg/dL)	0.6 ± 0.2	0.6 ± 0.3	0.6 ± 0.2	0.6692
Albumin (g/dL)	4.1 ± 0.3	4.3 ± 0.2	4.3 ± 0.2	0.0037
ALT (U/L)	22 ± 18	19 ± 9	19 ± 10	0.6414
GGT (U/L)	45 ± 69	33 ± 27	46 ± 41	0.3001
ALP (U/L)	244 ± 66	233 ± 87	215 ± 57	0.0463
BUN (mg/dL)	15.4 ± 3.8	14.6 ± 3.3	13.6 ± 3.7	0.0501
Cr (mg/dL)	0.74 ± 0.19	0.69 ± 0.14	0.72 ± 0.20	0.1558
Hematology				
Hemoglobin (g/dL)	13.4 ± 1.3	13.7 ± 1.2	13.6 ± 1.3	0.3195
Platelets (10^4^/μL)	21.0 ± 5.4	22.7 ± 5.5	23.2 ± 5.3	0.0033
Coagulation				
PT (%)	95.3 ± 13.1	92.9 ± 13.0	93.5 ± 11.8	0.5096
Fibrinogen (mg/dL)	268 ± 65	284 ± 69	279 ± 67	0.3484
TAT (ng/mL)	1.81 ± 1.37	1.08 ± 0.50	1.27 ± 0.86	0.0006
D-dimer (μg/mL)	0.99 ± 0.96	0.49 ± 0.33	0.58 ± 0.35	<0.0001
FDP (μg/mL)	1.96 ± 1.42	1.37 ± 0.38	1.47 ± 0.51	0.0002
Serology				
M2BPGi (COI)	0.72 ± 0.37	0.48 ± 0.22	0.60 ± 0.30	0.0003
AFP (ng/mL)	3.5 ± 1.5	3.6 ± 1.6	3.5 ± 1.5	0.9777
PIVKA-II (mAU/mL)	20.4 ± 6.2	20.8 ± 5.6	19.8 ± 5.5	0.5597
Ultrasound findings				
Tumor size (mm)	30.8 ± 22.8	14.1 ± 7.9	18.6 ± 10.4	<0.0001
Echo pattern				<0.0001
Homogeneous type (*n*)	47 (57.3%)	59 (89.4%)	44 (69.8%)	
Mixed type (*n*)	35 (42.7%)	7 (10.6%)	19 (30.2%)	
Portal vein diameter (mm)	11.0 ± 2.2	10.5 ± 2.1	11.2 ± 1.8	0.0219
Spleen index (mm^2^)	1273 ± 525	1180 ± 469	1432 ± 554	0.0143

Values are presented as means ± standard deviation or numbers (%). ALT, alanine aminotransferase; GGT, γ-glutamyl transpeptidase; ALP, alkaline phosphatase; BUN, blood urea nitrogen; Cr, creatinine; PT, prothrombin time; TAT, thrombin–antithrombin III complex; FDP, fibrin and fibrinogen degradation products; M2BPGi, Mac-2 binding protein glycosylation isomer; AFP, α-fetoprotein; PIVKA-II, protein induced by vitamin K absence or antagonist-II.

**Table 4:** Comparison between the first and last values for the follow-up period among each group of changes in size in 211 patients with hepatic hemangiomas.

	Decrease (*n* = 82)			No Change (*n* = 66)			Increase (*n* = 63)		
Parameters	First	Last	*p* Value	First	Last	*p* Value	First	Last	*p* Value
Tumor size (mm)	30.6 ± 22.8	23.3 ± 20.0	0.0054	14.1 ± 7.9	13.8 ± 8.3	0.4412	18.6 ± 10.4	24.5 ± 13.2	0.0034
Biochemistry									
Total bilirubin (mg/dL)	0.6 ± 0.2	0.6 ± 0.2	0.2129	0.6 ± 0.3	0.6 ± 0.2	0.2749	0.6 ± 0.2	0.6 ± 0.2	0.0539
Albumin (g/dL)	4.1 ± 0.3	4.1 ± 0.3	0.4147	4.3 ± 0.2	4.3 ± 0.2	0.1409	4.3 ± 0.2	4.3 ± 0.2	0.0647
ALT (U/L)	22 ± 18	21 ± 10	0.8174	19 ± 9	21 ± 10	0.0196	19 ± 10	21 ± 10	0.0083
GGT (U/L)	45 ± 69	47 ± 58	0.6030	33 ± 27	38 ± 32	0.0137	46 ± 41	49 ± 45	0.4218
ALP (U/L)	244 ± 66	265 ± 65	0.0031	233 ± 87	241 ± 85	0.0857	215 ± 57	227 ± 57	0.1314
Hematology									
Hemoglobin (g/dL)	13.4 ± 1.3	13.4 ± 1.3	0.3156	13.7 ± 1.2	13.9 ± 1.1	0.4698	13.6 ± 1.3	13.7 ± 1.5	0.1506
Platelets (10^4^/μL)	21.0 ± 5.4	21.0 ± 5.6	0.6953	22.7 ± 5.5	21.1 ± 4.6	0.0020	23.2 ± 5.3	21.8 ± 4.7	0.0064
Coagulation									
PT (%)	95.3 ± 13.1	99.0 ± 12.6	0.2421	92.9 ± 13.0	91.6 ± 10.0	0.3815	93.5 ± 11.8	94.7 ± 11.1	0.2745
Fibrinogen (mg/dL)	268 ± 65	281 ± 54	0.4427	284 ± 69	272 ± 69	0.3506	279 ± 67	264 ± 55	0.0154
TAT (ng/mL)	1.81 ± 1.37	1.39 ± 1.00	0.0455	1.08 ± 0.50	1.11 ± 0.67	0.5944	1.27 ± 0.86	1.72 ± 1.13	0.0102
D-dimer (μg/mL)	0.99 ± 0.96	0.67 ± 0.58	<0.0001	0.49 ± 0.33	0.46 ± 0.31	0.5170	0.58 ± 0.35	0.73 ± 0.38	0.0003
FDP (μg/mL)	1.96 ± 1.42	1.66 ± 0.94	0.0003	1.37 ± 0.38	1.44 ± 0.49	0.5159	1.47 ± 0.51	1.64 ± 0.74	0.0129
Serology									
M2BPGi (COI)	0.72 ± 0.37	1.05 ± 0.49	<0.0001	0.48 ± 0.22	0.56 ± 0.28	0.0610	0.60 ± 0.30	0.53 ± 0.24	0.0438
AFP (ng/mL)	3.5 ± 1.5	3.9 ± 1.8	0.0319	3.6 ± 1.6	3.9 ± 1.6	0.0221	3.5 ± 1.5	3.6 ± 1.7	0.8687
PIVKA-II (mAU/mL)	20.4 ± 6.2	21.7 ± 7.5	0.3236	20.8 ± 5.6	21.1 ± 5.8	0.1656	19.8 ± 5.5	20.5 ± 6.5	0.2711
Ultrasound findings									
Portal vein diameter (mm)	11.0 ± 2.2	11.1 ± 2.3	0.3156	10.5 ± 2.1	10.5 ± 1.8	0.9435	11.2 ± 1.8	11.1 ± 2.1	0.5667
Spleen index (mm^2^)	1273 ± 525	1306 ± 515	0.9915	1180 ± 469	1162 ± 473	0.7020	1432 ± 554	1409 ± 513	0.8461

Values are presented as means ± standard deviation or numbers (%). ALT, alanine aminotransferase; GGT, γ-glutamyl transpeptidase; ALP, alkaline phosphatase; BUN, blood urea nitrogen; Cr, creatinine; PT, prothrombin time; TAT, thrombin–antithrombin III complex; FDP, fibrin and fibrinogen degradation products; M2BPGi, Mac-2 binding protein glycosylation isomer; AFP, α-fetoprotein; PIVKA-II, protein induced by vitamin K absence or antagonist-II.

**Table 5:** Comparison of M2BPGi values among the different age groups under the specified categories in 321 patients initially diagnosed with hepatic hemangiomas.

	>60 y	40–60 y	<40 y	*p* Value
All patients (*n* = 321)	0.64 ± 0.33 (*n* = 105)	0.53 ± 0.29 (*n* = 156)	0.41 ± 0.18 (*n* = 60)	0.00002
Liver disease (−) (*n* = 274)	0.61 ± 0.33 (*n* = 87)	0.50 ± 0.28 (*n* = 128)	0.41 ± 0.19 (*n* = 59)	0.00013
Liver disease (+) (*n* = 47)	0.77 ± 0.32 (*n* = 18)	0.65 ± 0.28 (*n* = 29)		0.157
Small (*n* = 211)	0.59 ± 0.28 (*n* = 64)	0.46 ± 0.24 (*n* = 100)	0.36 ± 0.13 (*n* = 47)	0.00002
Medium (*n* = 83)	0.63 ± 0.41 (*n* = 30)	0.60 ± 0.31 (*n* = 42)	0.55 ± 0.22 (*n* = 11)	0.713
Large (*n* = 27)	0.89 ± 0.23 (*n* = 12)	0.82 ± 0.31 (*n* = 13)	0.80 ± 0.14 (*n* = 2)	0.235

All data represent values at the time of study enrollment. Data are expressed as M2BPGi values (means ± standard deviation) and number of patients. Only patients with chronic liver disease as the underlying disease were classified into two groups: >60 years and ≤60 years, since there were no patients aged <40 years. All patients, presence of liver disease (liver disease (−) or (+)), and hemangioma size (small, medium, or large) were included as categories.

## Data Availability

The data presented in this study are available upon request from the corresponding author.

## Abbreviations

PT	Prothrombin time
TAT	Thrombin–antithrombin III complex
FDP	Fibrin and fibrinogen degradation products
US	Ultrasonography
CT	Computed tomography
AFP	α-fetoprotein
PIVKA-II	Protein induced by vitamin K absence or antagonist-II
M2BPGi	Mac-2 binding protein glycosylation isomer
KMS	Kasabach–Merritt syndrome
